# Relating destabilizing regions to known functional sites in proteins

**DOI:** 10.1186/1471-2105-8-141

**Published:** 2007-04-30

**Authors:** Benoît H Dessailly, Marc F Lensink, Shoshana J Wodak

**Affiliations:** 1 Service de Conformation des Macromolécules Biologiques, Centre de Biologie Structurale et Bioinformatique, CP 263, Université Libre de Bruxelles (U.L.B), Bld. du Triomphe B-1050, Bruxelles, Belgium; 2 Structural Biology and Biochemistry Program, Hospital for Sick Children, 555 University Avenue, Toronto, Ontario M5G 1X8, Canada

## Abstract

**Background:**

Most methods for predicting functional sites in protein 3D structures, rely on information on related proteins and cannot be applied to proteins with no known relatives. Another limitation of these methods is the lack of a well annotated set of functional sites to use as benchmark for validating their predictions. Experimental findings and theoretical considerations suggest that residues involved in function often contribute unfavorably to the native state stability. We examine the possibility of systematically exploiting this intrinsic property to identify functional sites using an original procedure that detects destabilizing regions in protein structures. In addition, to relate destabilizing regions to known functional sites, a novel benchmark consisting of a diverse set of hand-curated protein functional sites is derived.

**Results:**

A procedure for detecting clusters of destabilizing residues in protein structures is presented. Individual residue contributions to protein stability are evaluated using detailed atomic models and a force-field successfully applied in computational protein design. The most destabilizing residues, and some of their closest neighbours, are clustered into destabilizing regions following a rigorous protocol. Our procedure is applied to high quality *apo*-structures of 63 unrelated proteins. The biologically relevant binding sites of these proteins were annotated using all available information, including structural data and literature curation, resulting in the largest hand-curated dataset of binding sites in proteins available to date. Comparing the destabilizing regions with the annotated binding sites in these proteins, we find that the overlap is on average limited, but significantly better than random. Results depend on the type of bound ligand. Significant overlap is obtained for most polysaccharide- and small ligand-binding sites, whereas no overlap is observed for most nucleic acid binding sites. These differences are rationalised in terms of the geometry and energetics of the binding site.

**Conclusion:**

We find that although destabilizing regions as detected here can in general not be used to predict binding sites in protein structures, they can provide useful information, particularly on the location of functional sites that bind polysaccharides and small ligands. This information can be exploited in methods for predicting function in protein structures with no known relatives. Our publicly available benchmark of hand-curated functional sites in proteins should help other workers derive and validate new prediction methods.

## Background

Available three-dimensional structures of proteins of unknown biological role are rapidly increasing as a result of structural genomics initiatives [1,2]. This prompted the development of methods for annotating protein structures at the residue level and inferring binding sites using information from related proteins [3-5]. A common approach to detect functional sites in proteins has been to identify evolutionarily conserved residues that are spatially contiguous in the protein structure [6-9]. This approach has lately been extended and integrated with detailed analyses of structural features, related protein structures and sequence information by several groups [10-13]. But the lack of related proteins, a common occurrence with structural genomics targets, hinders the wide applicability of many of these methods [14]. In addition, there are now many examples where above-average sequence variability rather than sequence conservation is associated with functional regions [15,16].

For these reasons, methods capable of identifying functional residues in absence of information on conserved residues [17] have attracted considerable attention. Available methods of this type are based on the detection of particular geometrical features in the protein structure, such as clefts [18], proximity of residues to the protein center [19], mutual spatial proximity of residues [20], or spatial motifs such as the well-known catalytic triad in serine proteases [21,22]. More recently, methods using a combination of evolutionary, geometrical and stability-related information to identify functional residues have also been proposed [23,24].

There are good indications that evolution often optimizes functional properties at the expense of thermodynamic stability. Site-directed mutagenesis of the catalytic residues in T4 lysozyme yields inactive mutant proteins that are more stable than the wild-type [25], indicating that the catalytic residues destabilize the wild-type enzyme. Similar conclusions were drawn from mutagenesis experiments on other proteins [26-30]. In particular, the catalytic power of enzymes is believed to result from the presence of specific constellations of polar residues in the active site, which can introduce either electrostatic [31] or steric [32,33] strain into the folded protein conformation in absence of the bound ligand [34]. Related to this observation is the recent finding that residues in left handed helices, which occur rarely in proteins, are often important for function [35].

Further evidence that protein sequences may not be optimized for protein stability has been provided by studies using computational protein design procedures. Those procedures select sequences that optimize the stability of a given protein three dimensional structure. They were recently shown to generate native-like sequences in the protein core but not on the surface, suggesting that surface residues may be selected primarily for functional reasons at the expense of stability [36].

Following this reasoning, several studies have shown that functional sites in protein structures can be detected by identifying residues positioned in unfavorable or unusual energetic environments. This includes the analysis of ionisable groups with perturbed titration curves [37] and the use of continuum electrostatics methods for the identification of polar residues engaged in unfavorable electrostatic interactions [38] in enzyme active sites [38-40]. Binding sites in proteins were also shown to consist of neighbouring regions of low and high stability [41].

A major challenge for functional site prediction methods in proteins is their validation against a benchmark set of known functional sites. Such benchmark must be large and diverse enough so as to cover many types of functional sites. These sites should furthermore be described in a standard fashion and this description should be based on all available information (structural, biochemical, site-directed mutagenesis etc.). Unfortunately, such benchmarks are still unavailable, although efforts are currently in progress to address this issue. Resources like the Catalytic Site Atlas [42] are very helpful, but are limited to catalytic residues in enzymes. They hence lack information on non-catalytic ligand-binding residues or other types of functionally important residues. Resources such as PdbSum [43], Pdb SITE records [44] or SwissProt [45] also provide useful information, but only for a subset of the entries. The BIND database [46] provides annotations for residues involved in ligand binding and different types of function, but makes no distinction between biologically relevant association modes and non-relevant ones. Other structure-based binding site databases suffer from that same limitation, and other issues that cannot be addressed without manual verification, such as inclusion of residues known to be important for function from non-structural evidence [47,48].

Providing a comprehensive and relevant functional site benchmark for proteins is not straightforward and reflects the difficulty to define what a functional site is. Where should one draw the limit? Should residues important for maintaining the native 3D structure or for enabling conformational changes, both of which may be required for function, also be considered as functional residues? Even when focusing on ligand binding alone, important choices need to be made in defining the ligand binding residues. Are those the residues that are directly involved in non-bonded interactions with the ligand in the *holo*-protein, or should one include other residues in the neighbourhood? One may choose to define ligand binding residues as those contributing significantly to the protein-ligand binding free energy. But this may likewise require the consideration of residues remote from the binding site, which might be involved in electrostatic steering effects [49,50]. Waiting for these issues to be addressed, functional site predictions are currently validated against information that is either approximate or incomplete. Some methods have been validated against sets of functional residues defined on the basis of thorough literature curation, but those are generally restricted to a very small numbers of proteins, and the definitions used are somewhat ad-hoc (*e.g*. [7]).

This paper presents a procedure for detecting destabilizing regions in protein three-dimensional structures solely on the basis of objective energetic criteria. The correspondence between these regions and known functional sites is quantitatively evaluated in order to assess the effectiveness of energetic criteria alone in functional site prediction. Our analysis focuses entirely on ligand-binding sites. To enable adequate validation we build a benchmark of 74 such binding sites from a non-redundant set (with sequence identity of at most 25%) of 63 proteins having a high quality crystal *apo*-structure, and at least one characterised binding site. These are defined here using a set of objective criteria and information extracted from the 3D structures and from a comprehensive analysis of the associated literature.

In our procedure the contribution of each residue to the protein folding free energy (*e.g*. its stability) is evaluated using an all-atom force-field developed previously for protein design applications [36,51]. Residues providing destabilizing free energy contributions are identified and grouped together to yield the destabilizing regions in a stepwise protocol, which takes into account their proximity in the 3D structure and the level of their destabilizing contribution. This protocol is governed by 4 adjustable parameters, which have straightforward physical meanings. These parameters are adjusted so as to optimize the overlap between the identified destabilizing regions and known binding sites in a set of 7 proteins (the learning set), which are unrelated (< 25% sequence identity) to the set of 63 proteins used for the analysis (the test set).

A systematic comparison between the destabilizing regions identified by our procedure in the 63 *apo*-protein structures and the known ligand binding sites reveals that their overlap is on average limited, but significantly better than random. A statistically significant overlap between the two types of regions (destabilizing and binding sites) is obtained in 77% of the proteins in which destabilizing regions are detected. Most interestingly, our study shows that the extent of overlap largely depends on the type of ligand whose binding site is being considered. Largest overlaps are obtained for sites binding small ligands and polysaccharides, while very poor overlap is almost systematically obtained with nucleic acid-binding sites. These differences are rationalized in terms of the geometric and energetic properties of the various binding sites, and the potential of using energetic criteria such as those proposed here for the prediction of functional sites in solved protein structures with no known relatives is discussed. The software DESITE for identifying destabilizing regions in protein structure is available upon request.

## Results

### Functional sites in proteins

In order to evaluate the degree of overlap between the destabilizing regions identified with our procedure and the regions that actually mediate function in the proteins of interest, an objective and unified description of the latter regions, termed here *functional sites *is required. To derive such description a detailed analysis that combined information from PDB entries and from biochemical and mutagenesis data extracted from the literature (see Methods) was conducted on the 63 proteins of our test set (see [52]).

A functional site was defined as a group of residues. In the vast majority of the cases the defined groups represent residues involved in ligand binding, where the ligands encompass molecules of different types and sizes. The defined sites are therefore strictly speaking ligand-binding sites. Hence residues not directly involved in ligand binding, but required for maintaining the stability of the native conformation or for enabling conformational changes required for function are not explicitly considered as being part of functional sites.

Table 1 summarizes the salient features of the characterized sites by protein and ligand type. Further details on the properties of each binding site and the full list of residues in the sites can be found at [53]. For all homo-multimers in the dataset, equivalent copies of the binding sites occur in the different subunits, but only a single copy is discussed here.

**Table 1:** Properties of known binding sites of the dataset proteins.

Pdb id^*a*^	Holo-pdb ids^*b*^	N res^*c*^	F res^*d*^	ASA^*e*^	F ASA^*f*^	Cleft^*g*^
**Small**						
1e1a		13	4.2	272	2.1	T
1e3f	1bm7, 1e4h, 1e5a, 1eta, 1tha	8	6.9	193	1.0	T
1gu7	1guf, 1n9g	31	8.5	1158	3.7	T
1gud	1rpj	24	8.3	976	7.4	T
1gus	1gug, 1gun, 1guo	4	6.0	119	0.9	F
1gus^*h*^	1gug, 1gun, 1guo	14	20.9	110	0.8	F
1gxy	1og1, 1og3, 1og4	24	10.8	947	8.7	T
1hf8	1hfa, 1hg2, 1hg5	4	1.5	329	1.4	F
1hhq	1hiy, 1b4s, 1b99, 1bux	17	11.3	1006	2.8	T
1is5	1is3, 1is4, 1is6	22	16.4	663	3.0	T
1jcf	1jcg	34	10.1	820	5.6	F
1odl	1odi, 1odj	25	10.7	339	0.8	T
1ofn	1oi6	15	7.4	669	4.0	T
1tm2	1tjy	19	6.1	437	3.2	T
1upq	1upr	12	11.2	783	11.9	T
1usg	1usk, 1usi	15	4.3	268	1.0	T
1usl	2bes, 2bet	18	11.5	505	3.9	T
1w1h	1w1d, 1w1g	10	6.6	560	6.2	T
1w2i	1w2i	8	8.9	450	5.0	F
1w37	1w3i, 1w3n, 1w3t	12	4.1	107	0.3	T
1y2t	1y2x, 1y2w	27	19.0	1191	5.6	F
						
**Polysaccharide**						
1nof		12	3.1	471	3.2	T
1o88		15	4.2	472	3.5	T
1ob0	1e3z	41	8.5	1572	8.9	T
1ogb	1e6n, 1e6r, 1h0g, 1h0i, 1ogg	16	3.2	565	1.5	T
1qhz	1qi2, 8a3h, 4a3h, 1e5j, 1qi0	14	4.6	590	5.2	T
1qjv		10	2.9	265	1.8	T
1uuq	1uz4	16	3.9	256	1.7	T
1w0n	1ux7	8	6.7	642	11.2	T
1w6z	1sf7, 1sfb, 1sfg	20	15.5	891	13.6	T
1w9s	1w9t, 1w9w	12	9.0	376	5.9	F
						
**Peptide**						
1c7k		9	6.8	275	4.2	T
1e5t	1e8m, 1e8n, 1o6g, 1qfs, 1uop	18	2.5	485	1.7	T
1ea7		7	2.3	97	0.9	T
1gt9	1gtj, 1gtl	21	5.9	450	3.4	F
1kl4	1hqq, 1kl3, 1kl5, 1rsu	17	14.2	854	4.2	T
1oes	1g1f, 1g1g, 1g1h, 1ptt, 1ptu	16	5.7	885	6.6	T
1r29	1r2b	29	23.8	1760	13.8	F
						
**Protein**						
1e3f	1qab, 1rlb	15	13.0	971	5.0	F
1e6l	1bdj	10	7.9	775	11.7	F
1e6l	1a0o, 1eay, 1ffg, 1ffs, 1ffw	15	11.8	1212	18.4	T
1eao	1e50, 1h9d	26	22.8	1819	28.2	F
1f2x		12	9.5	604	5.2	F
1gcp	1gcq	21	31.3	1405	33.2	F
1gqv	2bex	36	26.7	2246	28.9	F
1obq	1gka	22	12.2	1023	6.1	T
1sif	1cmx, 1fxt, 1nbf, 1otr, 1q5w, 1s1q, 1uzx	14	19.7	937	21.7	T
1tgr	1h59	15	28.8	1236	29.5	T
1uns	1jck	19	8.1	1620	13.5	F
1uns	1jwm	20	8.5	1386	11.6	F
1uol	1gzh, 1kzy	18	9.2	1301	13.1	F
1uq4	2aai	42	16.0	2796	21.6	F
1w53		12	14.3	770	8.8	T
						
**Nucleic acid**						
1e7l		8	5.1	352	2.0	T
1eao	1h9d, 1hjb	18	15.8	1375	21.3	F
1gqv	1hi3, 1hi4, 1hi5	9	6.7	245	3.2	T
1gv2	1h88, 1h89, 1mse	30	29.1	2289	30.2	T
1o7i		5	4.3	497	7.4	F
1okb	1emh, 1emj, 1q3f, 1ssp, 2ssp, 4skn	30	13.5	1539	15.0	T
1uol	1tsr, 1tup	19	9.7	1229	12.4	F
1uq4	1apg, 1br5	17	6.5	364	2.8	T
1utx		10	15.2	691	9.0	F
1vyi		10	9.0	1009	15.0	F
						
**Lipid**						
1obq	1h91, 1i4u, 1s2p, 1s44	21	11.6	350	2.1	T
1qmd		14	3.8	465	2.9	F
						
**Metal**						
1e6l	1chn, 1ymv	7	5.5	350	5.3	T
1qmd	1kho	6	1.6	67	0.4	T
**Peptide-Protein**						
1mix	1mk7, 1mk9	26	12.6	1526	11.9	F
						
**Small-Metal**						
1h1y		19	8.6	365	2.2	T
1h6l	2poo, 1h6l	16	4.5	627	4.4	T
1oid	1ho5, 1hp1, 1hpu	19	3.6	872	3.9	F
						
**Polysaccharide-Metal**						
1gkb	1bxh, 1cjp, 1c57, 1ces, 1dq1, 1gkb, 3cna, 3enr	19	8.0	586	1.8	F
**Lipid-Metal**						
1umv	1pob, 1umv, 1c1j	17	13.9	492	3.8	T
						
**Protein-Metal**						
1o6v	1o6s	49	10.6	2224	11.3	F

Properties of known binding sites of the dataset proteins. Binding sites are classified according to their type of ligand. The last 5 categories refer to binding sites where 2 types of ligand can bind.

^*a *^Pdb identifier of structure used for energy calculations.

^*b *^Pdb identifiers of the structures of the protein-ligand complex used to define the binding site.

^*c *^Number of residues in binding site.

^*d *^Fraction of protein residues in binding site (in %).

^*e *^Total ASA of binding site residues.

^*f *^Fraction of protein ASA in binding site (in %).

^*g *^True (T) if binding site sits in a cleft, False (F) otherwise.

^*h *^1gus appears twice here because it has 2 distinct binding sites for small ligands. The same observation applies to 1e6l and 1uns that have 2 distinct binding sites for different proteins.

The 63 proteins of our dataset were found to contain a total of 74 binding sites, with 9 proteins containing 2 binding sites, and one protein (CheY) with 3 binding sites. The analyzed proteins contain 10 nucleic acid-binding sites, 11 polysaccharide binding sites, 17 interaction sites with other proteins, 8 peptide binding sites, 3 for lipids, 8 for metal ions, and 24 small ligand-binding sites. Seven sites were found to bind multiple ligands. The size of the binding sites, expressed in terms of the number of residues per site copy, varies from 4 to 49 residues, with an average of 17 residues. The fraction of the total number of residues contained in individual binding sites ranges between 1.5% and 31.3% (with an average of ~10%). But most binding sites (67/74) have a small relative size of less than 2% of the total number of residues in the protein.

**Figure 1:**
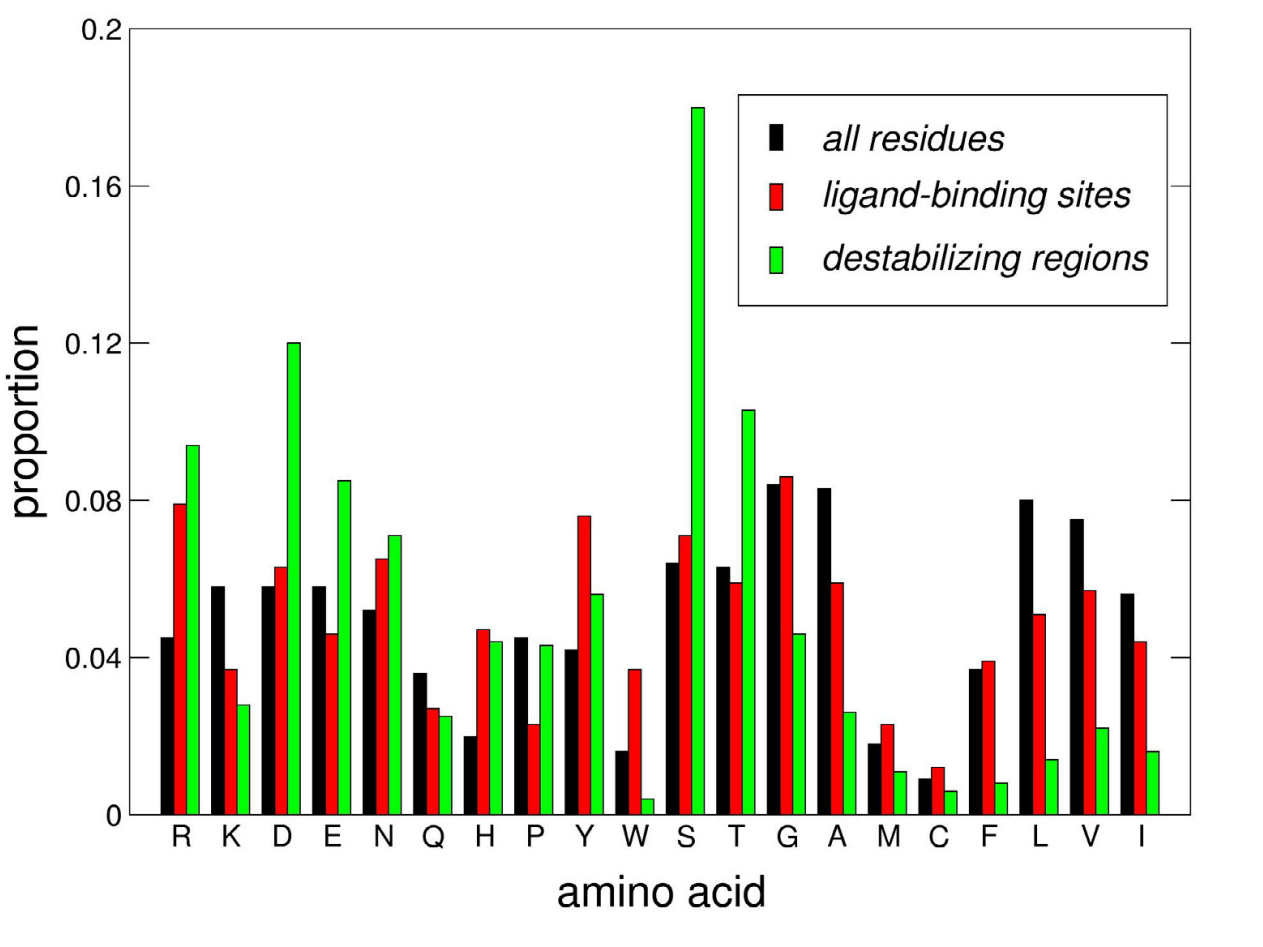
**Amino acid composition in proteins of our dataset, functional sites and destabilizing regions**. Mean proportions of residue types for all residues in the dataset, binding sites residues and destabilizing regions residues. Residues are sorted with increasing hydrophobicity according to Kyte-Doolittle scale [76].

The average amino acid composition found in binding sites is illustrated in Fig. 1. In comparison to the amino acid composition of the complete proteins of our set (Fig. 1), binding sites are enriched in aromatic residues (W, Y), H, R, and N, and to a lesser extent in M and D. On the other hand these sites are depleted in P, K, E and the aliphatic residues (A, V, L, I). The lower occurrence of lysine residues in binding sites, as compared with other polar residues, thus follows the trend observed previously for protein-protein interfaces [54] and catalytic sites [55]. The enrichment in aromatic and polar residues such as H, T, R, and N most probably reflects the hydrogen bonding potential of the corresponding side chains, with the aromatics mostly occuring in sugar-binding sites. The frequent occurrence of Arg residues has previously been reported in interfaces of protein complexes [56].

Binding sites have been reported to often occur in large clefts at the protein surface [18]. This is particularly frequent for sites that bind small ligands as it enables the protein to surround such ligands almost completely in order to minimize contact with the solvent [57]. To investigate the extent to which this was also the case for the binding sites identified here, we checked whether the sites were located in one of the 4 largest clefts identified by SURFNET [18], following a set of recent rules used in conjunction with this program [58]. We consider a binding site to be part of one of these clefts if at least 75% of its residues are cleft residues as well. Residues lining SURFNET clefts are defined with the MASK program (provided with SURFNET) using default values [59]. Inspection of Table 1 reveals that out of the 74 binding sites in our dataset, 45 are located in such clefts, including the majority of the sites that bind small ligands (17/24) and polysaccharides (9/11). The frequency is lower for other ligand types, particularly for peptide and protein binding sites (5/17). As a consequence of their preferred location in clefts, residues belonging to functional sites generally have lower solvent accessibilities (25.2 ± 25.5%) than non-functional surface residues (40.3 ± 25.8%), with about 25% of residues in these sites being completely buried. It should also be noted that the largest cleft identified by SURFNET is usually much larger than any of the functional sites defined here (which comprise 17 residues on average), with the largest clefts containing at least 20 residues and often as many as several hundreds (see [60]). The probability that the overlap of such large clefts with the much smaller functional sites might occur by chance can therefore be quite high (see discussion below on the predictive power of destabilizing regions).

### Destabilizing regions

#### Salient features

Destabilizing regions are defined as groups of spatially neighbouring residues whose contribution to the free energy of the native state is unfavorable (destabilizing). Such groups are identified by first computing the contributions of individual residues to the protein folding free energy, selecting the residues with the most unfavorable contributions and delimiting regions in the protein that contain a high density of such residues, as described in Methods.

Table 2 summarizes the salient features of the destabilizing regions identified in the 63 proteins of our dataset. They include the number of residues in each region, the fraction of the total number of protein residues found in the region, and whether the region maps into a large cleft. Further details for each site, including the identity of individual residues are given at [61].

**Table 2:** Properties of destabilizing regions detected in the dataset proteins.

Pdb id^*a*^	N Reg^*b*^	N res^*c*^	F res^*d*^	ASA^*e*^	F ASA^*f*^	Cleft^*g*^
1c7k	1	7	5.3	196	3.0	F
1e1a	1	9	2.9	331	2.5	T
	1	10	3.2	290	2.2	F
	1	19	6.1	256	1.9	F
1e3f	1	18	3.9	170	0.9	T
1e5t	1	18	2.5	450	1.6	T
	1	11	1.5	405	1.4	T
	1	4	0.6	143	0.5	T
1e6l	1	6	4.7	410	6.2	T
1ea7	1	4	1.3	207	1.9	T
	1	17	5.5	495	4.5	F
	1	7	2.3	546	4.9	F
	1	9	2.9	82	0.7	F
	1	4	1.3	196	1.8	T
1f2x	1	4	1.6	230	2.0	F
1gcp	1	10	14.9	644	15.2	F
1gkb	4	22	9.3	152	2.0	F
1gqv	1	8	5.9	573	7.4	F
1gt9	1	8	2.2	309	2.3	F
	1	10	2.8	114	0.9	F
	1	10	2.8	534	4.0	F
1gu7	2	6	1.6	434	2.8	T
	2	11	3.0	465	3.0	T
1gud	1	6	2.1	422	3.2	F
	1	20	6.9	571	4.4	T
	1	4	1.4	289	2.2	T
	1	6	2.1	234	1.8	F
1gus	4	13	12.9	262	7.6	T
1gxy	1	12	5.4	107	1.0	T
	1	6	2.7	386	3.5	T
1h1y	1	12	2.7	546	3.3	T
	2	9	4.1	179	2.2	T
1h6l	1	21	5.9	493	3.5	F
	1	4	1.1	195	1.4	T
1hf8	2	5	1.9	425	3.6	F
	2	18	6.8	816	6.8	T
1hhq	6	7	4.7	114	1.8	F
	6	13	8.7	748	12.6	T
1is5	4	4	3.0	185	3.2	T
1jcf	1	14	4.2	553	3.8	T
	1	5	1.5	256	1.7	F
	1	19	5.7	316	2.2	F
	1	8	2.4	662	4.5	T
1kl4	4	5	4.2	429	8.4	T
1mix	1	11	5.3	705	5.5	T
	1	6	2.9	408	3.2	F
	1	4	1.9	304	2.4	T
1nof	1	25	6.5	587	4.0	F
	1	6	1.6	255	1.7	F
	1	6	1.6	415	2.8	F
	1	6	1.6	144	1.0	F
1o6v	1	11	2.4	376	1.9	F
	1	11	2.4	679	3.5	F
	1	13	2.8	608	3.1	F
	1	5	1.1	315	1.6	F
1o7i	1	4	3.5	346	5.1	T
1o88	1	7	2.0	198	1.5	T
	1	5	1.4	296	2.2	F
	1	7	2.0	313	2.3	F
	1	4	1.1	211	1.6	F
	1	4	1.1	248	1.8	F
	1	7	2.0	190	1.4	T
1ob0	1	6	1.2	314	1.8	F
	1	7	1.5	226	1.3	T
	1	4	0.8	138	0.8	F
	1	7	1.5	93	0.5	T
1obq	1	4	1.1	217	1.3	T
	1	15	4.2	318	1.9	T
1odl	3	10	2.1	547	3.6	F
	6	12	5.1	276	3.6	T
	3	23	4.9	54	0.3	T
1oes	1	6	2.1	97	0.7	F
	1	5	1.8	105	0.8	T
	1	7	2.5	377	2.8	T
	1	9	3.2	423	3.1	F
1ofn	2	11	5.4	395	4.8	T
	2	9	4.4	324	3.8	T
1ogb	1	11	1.1	455	1.2	F
	1	15	1.5	149	0.4	F
	2	14	2.8	765	4.0	F
	2	4	0.8	236	1.2	F
1oid	1	4	0.8	291	1.3	F
	1	17	3.3	438	2.0	F
	1	14	2.7	641	2.9	F
	1	20	3.8	504	2.3	T
	1	5	1.0	325	1.5	F
1okb	1	4	1.8	300	2.9	F
	1	14	6.3	704	6.9	T
	1	6	2.7	329	3.2	T
1qhz	1	24	7.9	328	2.9	T
	1	5	1.7	170	1.5	F
1qjv	1	5	1.5	359	2.4	T
	1	33	9.6	1164	7.9	F
	1	7	2.0	165	1.1	T
1qmd	1	6	1.6	129	0.8	T
	1	4	1.1	319	2.0	T
	1	6	1.6	417	2.6	F
	1	21	5.7	828	5.2	T
1tm2	1	4	1.3	330	2.4	F
	1	22	7.0	663	4.9	T
1umv	2	5	4.1	233	3.6	T
1uns	1	4	1.7	246	2.1	T
	1	7	3.0	262	2.2	F
1uol	1	7	3.6	368	3.7	F
1uq4	1	5	1.9	251	1.9	T
1usg	1	30	4.3	715	2.7	T
	2	13	3.8	340	2.6	T
1usl	2	8	5.1	304	4.8	F
1uuq	1	13	3.2	50	0.3	F
	1	5	1.2	53	0.3	T
	1	6	1.5	182	1.2	F
	1	11	2.7	356	2.3	F
1w0n	1	10	8.3	455	7.9	F
1w1h	1	7	4.6	431	4.8	T
1w2i	2	13	14.4	750	16.6	T
1w37	8	7	4.8	131	2.4	F
	8	8	5.5	83	1.6	F
1w6z	1	14	10.9	592	9.0	F
1y2t	1	8	1.4	274	1.3	T
	2	8	2.8	1	0.0	F
	4	10	7.0	415	7.6	T

^*a *^Pdb identifier of structure used for energy calculations.

^*b *^Number of equivalent destabilizing regions identified in the structure (relevant for multimers only).

^*c *^Number of residues in destabilizing region.

^*d *^Fraction of protein residues in destabilizing region (in %).

^*e *^Total ASA of destabilizing region residues.

^*f *^Fraction of protein ASA in destabilizing region (in %).

^*g *^True (T) if destabilizing region sits in a cleft, False (F) otherwise.

In homo-multimeric assemblies the described destabilizing regions represent the smallest of the equivalent destabilizing regions identified in different subunits, and can be considered as the common core of these regions. A similar procedure was applied to define the common core of intersecting regions between binding sites and destabilizing regions, in multimeric proteins (see below).

A total of 121 destabilizing regions are detected in the dataset, but none are found in 11 proteins (pdb ids 1utx, 1gv2, 1eao, 1e7l, 1vyi, 1w9s, 1upq, 1w53, 1tgr, 1r29, 1sif). The number of destabilizing regions per protein ranges between zero in these 11 cases and 6 in one protein. Their size varies from 4 to 33 residues, averaging around 10 residues, and most destabilizing regions (101/121) contain less than 15 residues. Residues in a single destabilizing region represent between <1% and ~15% of the total number of residues in the protein, with an average of ~4%. Less than half (59/121) of the destabilizing regions map into one of the 4 largest clefts in the protein.

The average amino acid composition of destabilizing regions is illustrated in Fig. 1, alongside of the composition in binding sites and in the full proteins of our dataset. Relative to the amino acid composition of the full protein, these regions are highly enriched in S and D, and to a lesser extent in E, R, T and H, and are largely depleted in the aliphatic residues (A, V, I, L), as well as in K, W, G, M and C. The amino acid composition of the destabilizing regions thus displays some similarities to the composition of the binding sites (low representation of lysines and aliphatic residues) but also differs from it, most notably by the lower content of aromatics, and much higher content of S, T, D and E. Overall, polar and charged residues account for more than 75% of the residues in the destabilizing regions.

#### Origins of the unfavorable energetic contributions

To gain insight into the origins of the unfavorable energy contributions of the so-called destabilizing residues, the contribution of individual residues to the folding free energy of the protein Δ*G*_*f *_is decomposed into individual terms as follows:

Δ*G*_*f *_= Δ*G*_*vdw *_+ Δ*G*_*elec *_+ Δ*G*_*solvation*_

**Figure 2:**
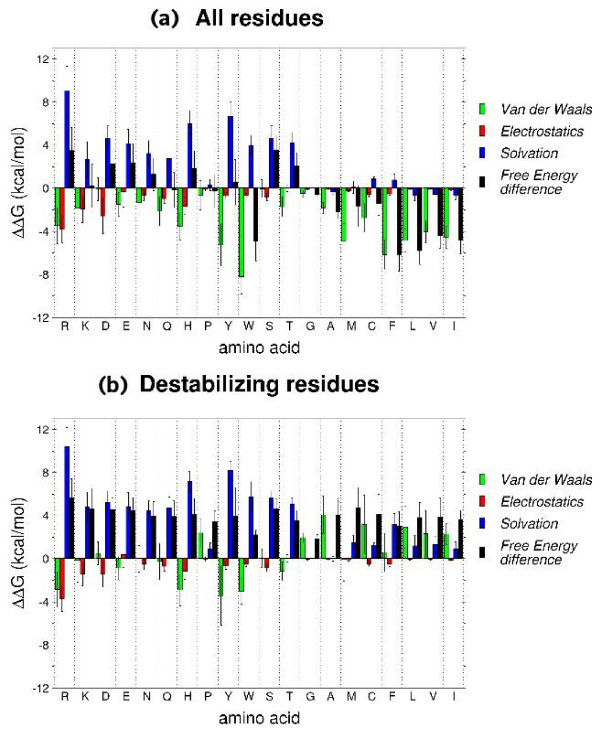
**Average Van der Waals, electrostatics and solvation contributions, and total free energy difference, for each residue type**. Average values of the Van der Waals, electrostatics and solvation terms, and of the total free energy difference, for each residue type, computed over (*a*) all residues in the dataset, and (*b*) destabilizing residues. Standard deviations are indicated as error bars. Residues are sorted with increasing hydrophobicity according to Kyte-Doolittle scale [76].

Where Δ*G*_*vdw*_, Δ*G*_*elec *_and Δ*G*_*solvation *_are respectively the differences in Van der Waals, electrostatic and solvation free energies between the folded state and the reference state for a given residue (see Methods). Figure 2 shows the average values and standard deviations for the different terms in Eq. 1 and the total free energy difference, computed for the 20 amino acid types, of all the residues of our dataset (Fig. 2a) and of the residues identified as destabilizing by our analysis (Fig. 2b) (see Methods).

The polar residues in our dataset generally exhibit an unfavorable contribution to the solvation free energy difference (Fig. 2a) because their polar groups are often partially buried in the folded protein. However, with those groups often engaged in hydrogen bonds with neighbouring residues in the protein, the unfavorable effect of desolvation tends to be at least partially compensated by more favorable electrostatic and Van der Waals interactions made in the folded protein than in water. For K, N, Q and Y residues these compensatory effects roughly balance each other, yielding a net contribution to the folding free energy that is near zero (Fig. 2a). In R, D, E, H, S and T the unfavorable contribution due to desolvation is less effectively compensated by other terms, leading to a net destabilizing contribution. It is thus not surprising that these residues are significantly over-represented in destabilizing regions. For the hydrophobic residues desolvation generally results in a near zero or favorable contribution, whereas the contribution from Van der Waals interactions tends to be stabilizing, especially for F, Y and W, reflecting their tight packing in the folded state [62]. The values calculated for the electrostatic contribution are close to zero for most residue types, except for arginines where they seem to be greatly stabilizing.

For destabilizing residues (Fig. 2b) the contribution from desolvation is in general more destabilizing for all residues types, and the favorable contributions from the Van der Waals and electrostatic terms is in general weaker, leading to a net destabilizing effect overall. These trends are most salient for polar residues, which represent the major fraction of the destabilizing residues identified in our dataset. When hydrophobic residues are identified as destabilizing this is often due to the solvation and Van der Waals terms being unfavorable. In comparison to the hydrophobic residues in the full dataset whose contribution to the Van der Waals term is in general quite stabilizing, the absence of favorable Van der Waals contributions in destabilizing hydrophobic residues is particularly striking (Fig. 2a,b).

### Relation between destabilizing regions and known binding sites

In total, there are more destabilizing regions (121) than known binding sites (74), but destabilizing regions tend to be smaller, with ~10 residues on average *vs*. 17 in functional sites. This results in similar average numbers of residues in destabilizing regions and known binding sites, per protein (19 and 20 residues, respectively). The average fraction of protein residues located in binding sites (10%) is however much larger than that located in destabilizing regions (~4%) because several analyzed proteins with known binding sites do not contain destabilizing regions. We also see that known binding sites are more frequently located in large clefts than destabilizing regions (45/74 *vs*. 59/121).

#### Intersection of functional sites and destabilizing regions

Detected destabilizing regions and known binding sites show overlap in 45 out of the total of 63 proteins in our dataset. Residues shared by a known binding site and a destabilizing region constitute what we call here the Intersection Region (IR).

Table 3 lists the details of the overlaps, including the number of residues in the known binding site, in the destabilizing region, and in the IR. In the case of multimeric proteins, the listed numbers were computed considering all the subunits. In total, 60 IR's are identified and more than one IR's are found in 16 proteins. Several destabilizing regions overlap with the same known functional site in 14 proteins. In pectate lyase C ([PDB:lo88]), the polysaccharide-binding site overlaps with 3 different destabilizing regions. In crustacyanin ([PDB:1obq]) and phospholipase C ([PDB:1qmd]), 2 different binding sites overlap with a single destabilizing region. Twenty-six overlapping regions are located in small ligand-binding sites, 14 in polysaccharide-binding sites, 10 in protein-binding sites, 7 in peptide-binding sites, 6 in metal-binding sites, 2 in lipid-binding sites, and 1 in a nucleic acid-binding site.

**Table 3:** Details of the intersection between binding sites and destabilizing regions^*a*^.

Pdb id^*b*^	N site^*c*^	N des.^*d*^	N IR^*e*^	Sens.^*f*^	PPV^*g*^	Exp N IR^*h*^	P-value
**Small**							
1e1a	13	38	7	53.8	18.4	1.6	0.00023
1e3f	32	18	8	25.0	44.4	1.2	5.9e-06
1gu7	62	53	10	16.1	18.9	4.5	0.00999
1gud	24	36	14	58.3	38.9	3.0	1.5e-08
1gus	24	73	0	0.0	0.0	4.4	1.0
1gus^*i*^	84	73	41	48.8	56.2	15.2	6.2e-14
1gxy	24	18	7	29.2	38.9	1.9	0.00100
1hf8	8	46	0	0.0	0.0	0.7	1.0
1hhq	102	126	30	29.4	23.8	14.3	1.2e-05
1is5	88	16	16	18.2	100.0	2.6	8.2e-14
1jcf	34	46	12	35.3	26.1	4.7	0.00061
1odl	150	190	60	40.0	31.6	20.3	3.3e-18
1ofn	30	46	13	43.3	28.3	3.4	2.9e-06
1tm2	19	26	6	31.6	23.1	1.6	0.00232
1usg	30	56	14	46.7	25.0	2.4	5.6e-09
1usl	36	28	15	41.7	53.6	3.2	5.5e-09
1w1h	10	7	6	60.0	85.7	0.5	9.6e-08
1w2i	16	24	0	0.0	0.0	2.1	1.0
1w37	48	101	16	33.3	15.8	4.1	6.5e-07
1y2t	108	66	0	0.0	0.0	12.6	1.0
							
**Polysaccharide**							
1nof	12	43	7	58.3	16.3	1.4	7.3e-05
1o88	15	34	12	80.0	35.3	1.4	3.2e-11
1ob0	41	24	8	19.5	33.3	2.0	0.00037
1ogb	32	53	4	12.5	7.5	1.7	0.08496
1qhz	14	29	10	71.4	34.5	1.3	1e-08
1qjv	10	45	4	40.0	8.9	1.3	0.03039
1uuq	16	35	10	62.5	28.6	1.4	3.1e-08
1w0n	8	10	0	0.0	0.0	0.7	1.0
1w6z	20	14	6	30.0	42.9	2.2	0.00887
							
**Peptide**							
1c7k	9	7	0	0.0	0.0	0.5	1.0
1e5t	18	33	4	22.2	12.1	0.8	0.00748
1ea7	7	41	0	0.0	0.0	0.9	1.0
1gt9	21	28	4	19.0	14.3	1.6	0.07134
1kl4	68	22	12	17.6	54.5	3.1	5.6e-06
1oes	16	27	5	31.2	18.5	1.5	0.01154
							
**Protein**							
1e3f	60	18	0	0.0	0.0	2.3	1.0
1e6l	10	6	3	30.0	50.0	0.5	0.00632
1e6l	15	6	0	0.0	0.0	0.7	1.0
1f2x	24	4	0	0.0	0.0	0.4	1.0
1gcp	21	10	2	9.5	20.0	3.1	0.89027
1gqv	36	8	6	16.7	75.0	2.1	0.00465
1obq	44	19	11	25.0	57.9	2.3	9.6e-07
1uns	19	4	0	0.0	0.0	0.9	1.0
1uns	20	4	4	20.0	100.0	0.9	0.00852
1uol	18	7	2	11.1	28.6	0.7	0.12811
1uq4	42	5	4	9.5	80.0	0.8	0.00254
							
**Nucleic acid**							
1o7i	5	4	0	0.0	0.0	0.2	1.0
1okb	30	24	12	40.0	50.0	3.2	3.7e-06
1uol	19	7	0	0.0	0.0	0.7	1.0
1gqv	9	8	0	0.0	0.0	0.5	1.0
1uq4	17	5	0	0.0	0.0	0.3	1.0
							
**Lipid**							
1obq	42	19	7	16.7	36.8	2.2	0.00305
1qmd	14	37	4	28.6	10.8	1.4	0.04088
							
**Metal**							
1e6l	7	6	0	0.0	0.0	0.3	1.0
1qmd	6	37	1	16.7	2.7	0.6	0.47097
							
**Peptide-Protein**							
1mix	26	21	6	23.1	28.6	2.6	0.03234
							
**Small-Metal**							
1h1y	38	33	17	44.7	51.5	2.9	8.6e-12
2poo	16	25	10	62.5	40.0	1.1	2.8e-09
1oid	19	60	14	73.7	23.3	2.2	1.2e-10
							
**Polysaccharide-Metal**							
1gkb	76	88	48	63.2	54.5	7.0	1.4e-36
							
**Lipid-Metal**							
1umv	34	19	0	0.0	0.0	2.6	1.0
							
**Protein-Metal**							
1o6v	49	40	12	24.5	30.0	4.2	0.00032

**Small**				**34.4 (21.4)^*j*^**	**32.3 (25.5)^*k*^**		
**Polysaccharide**				**43.7 (27.3)**	**26.2 (17.4)**		
**Peptide**				**18.3 (14.0)**	**16.6 (16.7)**		
**Protein**				**13.0 (10.9)**	**36.2 (33.9)**		
**Nucleic acid**				**8.0 (17.9)**	**10.0 (22.4)**		
**Lipid**				**15.1 (14.4)**	**15.9 (18.9)**		
**Metal**				**35.7 (29.4)**	**25.3 (22.6)**		

^*a *^No destabilizing regions were detected in 11 entries of the dataset (1utx, 1gv2, 1eao, 1e7l, 1vyi, 1w9s, 1upq, 1w53, 1tgr, 1r29, 1sif) and these entries are not listed in this table.

^*b *^Pdb identifier of structure used for energy calculations.

^*c *^Number of residues in binding site.

^*d *^Number of residues in destabilising region.

^*e *^Number of residues in intersection region.

^*f *^Sensitivity (in %).

^*g *^Positive predictive value (in %).

^*h *^Expected number of residues in intersection region (see text).

^*i *^1gus appears twice here because it has 2 distinct binding sites for small ligands. The same observation applies to 1e6l and 1uns that have 2 distinct binding sites for different proteins.

^*j *^Average sensitivity (and standard deviation) for the given ligand type.

^*k *^Average PPV (and standard deviation) for the given ligand type.

The majority of the IR's (78%) are located in one of the four largest clefts of each protein. The number of residues per IR varies from 1 to 12, with an average of 5. Most contain less than 8 residues.

#### Can destabilizing regions be used to predict functional sites?

Using the results on the overlap between destabilizing regions and known functional sites listed in Table 3, we now evaluate the extent to which destabilizing regions, as identified here, can be used to predict functional sites in a protein structure in absence of prior knowledge.

To that end the sensitivity S and positive predictive value (PPV) of the destabilizing regions were computed. For a given protein, S_*obs *_is the fraction of residues in known binding sites that are also part of destabilizing regions; and PPV_*obs *_is the fraction of residues in destabilizing regions that is intersecting with known binding sites (see Methods). Since our procedure was not trained on our dataset but on a different group of 7 proteins, this dataset can be used to compute these quantities. The average values of S_*obs *_and PPV_*obs*_in the dataset are respectively 25.7% and 27.0%. This means that, on average, about one fourth of the residues in a given binding site are also part of destabilizing regions, and that a little more than one fourth of the residues in destabilizing regions are also part of binding sites. The observed S and PPV values for each protein in which destabilizing regions were identified are listed in Table 3.

Although this overlap is modest and would not allow to identify functional sites in a quantitative fashion, it is statistically significant. The number of overlapping residues expected by chance for each binding site-destabilizing region pair is computed using the hypergeometric distribution (see Methods). These expected numbers of intersecting residues were used to calculate the expected sensitivity (*S*_*exp*_) and PPV (*PPV*_*exp*_) values for each protein. The average *S*_*exp *_and *PPV*_*exp *_are 7.9% and 9.3%, respectively. A one-tailed Wilcoxon signed-rank test [63] was then applied to the full set of observed and expected sensitivity and PPV values, and indicates that *S*_*obs *_are significantly larger than *S*_*exp*_(*p *= 3.305*e *- 08) and that *PPV*_*obs*_are significantly larger than *PPV*_*exp*_(*p *= 4.091*e *- 07).

Taking a P-value threshold of 0.05 in considering an overlap as statistically significant, yielded statistically significant overlaps in 40 of the 52 proteins in which at least one destabilizing region has been identified. In other words, if destabilizing regions are identified in a protein, there is 77% (40/52) probability that at least one of these regions will display a significant overlap with at least one known binding site. In the following we describe how these significant overlaps are distributed amongst the functional sites associated with different types of bound ligands.

#### Overlap with destabilizing regions as a function of ligand types

**Figure 3:**
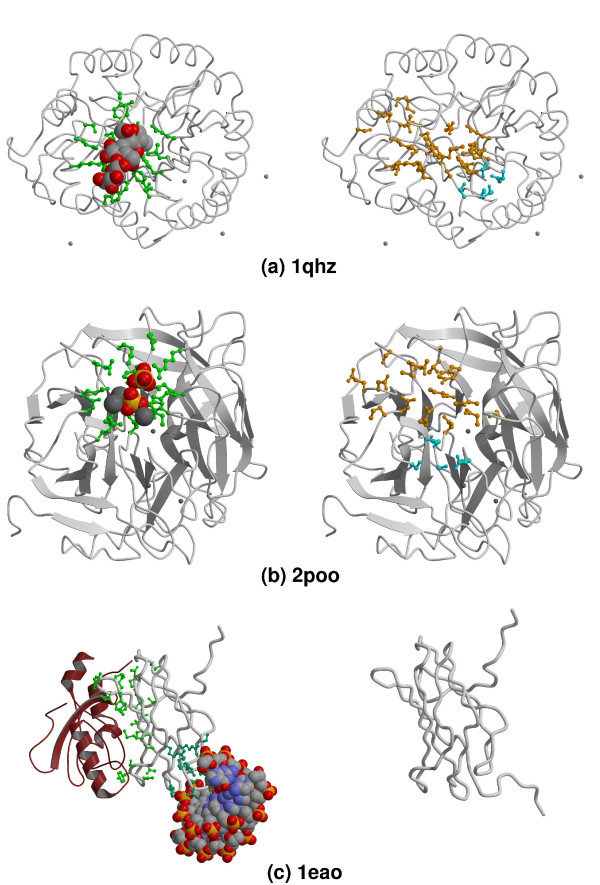
**Examples of known binding sites and destabilizing regions identified in 3 proteins**. Each protein is represented twice: its binding sites (residues colored green) and ligands (displayed and colored as cpk) are shown on the left panel, whereas destabilizing regions (residues colored orange or cyan) are shown on the right panel. Represented residues are all displayed as "balls-and-sticks". Ligands considered as biologically irrelevant are displayed on each panel as balls-and-sticks and colored cpk. Pdb ids used to reference subfigures are those used in the text and tables. (a) Endoglucanase B (Pdb id 1qi2  and 1qhz used for left and right panels, respectively), a protein with a polysaccharide-binding site. Backbone is displayed as coil and colored grey. PPV = 34.5%, Sensitivity = 71.4%. Two destabilizing regions are detected in this protein (one in orange and the other in cyan). (b) Phytase (Pdb id 1h6l and 2poo used for left and right panels, respectively), a protein with a small ligand-binding site. The backbone is displayed as cartoons and colored grey. PPV = 40.0%, Sensitivity = 62.5%. Two destabilizing regions are detected in this protein (one in orange and the other in cyan). (c) AML-1 (Pdb id 1h9d and 1eao used for left and right panels, respectively), a protein with a protein-binding site and a nucleic acid-binding site. The bound protein, CBF-*β*, is represented as cartoons and colored dark-red. AML-1 is displayed as coil and colored grey. No destabilizing region was detected in this protein. Figures 3 and 5 were drawn with Molscript [77] and rendered with Raster3D [78].

Figure 3 illustrates the comparison of binding sites with destabilizing regions as detected using our procedure. Results are shown for proteins that bind different types of ligands (polysaccharides, small ligands, nucleic acids and proteins).

The extent of overlap between destabilizing regions and functional sites varies significantly with the type of ligand binding to these sites (Fisher exact test [63]: contingency table is Table 4, *p *= 0.005). This is not too surprising given that the shape and composition of the functional site is in principle optimized to fit the type of ligand that binds to it, and that occurrence of destabilizing regions depends on shape and amino acid composition. The average sensitivity is highest (43.7%) for sites that bind polysaccharides and lowest (8%) for nucleic acid binding sites, whereas the average PPV ranges from 36.2% for protein binding sites to 10% for nucleic-acid binding sites (see Table 3).

**Table 4:** Overlap between destabilizing regions and binding sites according to ligand type

Ligand type	Nu	Po	Pr	Pe	Sm	Me	Li	Total
Sig. Overlap	1 (5.7)	8 (6.2)	7 (9.6)	5 (5.7)	19 (13.6)	5 (4.5)	2 (1.7)	47
No. Overlap	9 (4.3)	3 (4.8)	10 (7.4)	5 (4.3)	5 (10.4)	3 (3.5)	1 (1.3)	36

Total	10	11	17	10	24	8	3	83

Contingency table used to perform a Fisher exact test of homogeneity, among different categories of binding sites (based on ligand types), of the fraction of statistically significant overlaps (*i.e*. P-value ≤ 0.05, see Methods for meaning of the P-value) between destabilizing regions and known binding sites. Abbreviations used: Nu, nucleic-acid; Po, polysaccharide; Pr, protein; Pe, peptide; Sm, small compound; Me, metal ion; Li, lipid; Sig. Overlap, statistically significant overlap; No. Overlap, statistically non-significant or absent overlap. Calculated expected numbers of statistically significant overlaps are given between brackets, below the corresponding observed numbers.

Destabilizing regions identified here are reasonable predictors of binding sites for polysaccharides and small ligands, but very poor predictors of sites involved in nucleic acid binding, with the prediction performance for other types of sites being of intermediate reliability (see Tables 3 and 4). The better overlap with sites that bind small ligands and polysaccharides can be explained by the fact that many small ligands and polysaccharides bind to clefts enriched in polar and/or charged residues [57]. The polar residues in these sites therefore tend to be more buried than average, thereby providing a destabilizing contribution to the folding free energy in absence of the ligand [29]. The same applies to metal-binding sites located in deep clefts, with some exceptions however, as in phospholipase C ([PDB:1qmd]) where the zinc-binding site undergoes very large conformational change upon binding [64]. In internalin A ([PDB:lo6v]), CheY ([PDB:le6l) and endonuclease VII ([PDB:le7l), the metal ion binds in shallow clefts or flat surfaces where the residues have freedom to move to adapt to the absence of the ion.

In contrast, nucleic acids generally bind to larger regions with flat or convex surface shape. As a result, the binding site residues can be well solvated in absence of the bound ligand. Furthermore, nucleic acid binding regions usually include a sizable fraction of aliphatic and aromatic residues [65], which are poorly represented in the destabilizing regions identified here.

The overlap with lipid-binding sites, which are often located in deep clefts rich in hydrophobic residues, is in general rather limited. Significant overlap is however observed with sites in phospholipase C ([PDB:1qmd]) and crustacyanin C1 ([PDB:1obq]). These proteins bind polar heads carrying phospholipids, and the corresponding sites bury these heads inside polar clefts on the protein surface. It is these polar clefts that tend to overlap with the destabilizing regions identified in these proteins.

Protein and peptide binding sites are very diverse, and so is their overlap with destabilizing regions. Some functional sites or part of such sites are located in disordered regions and cannot be identified by our method (see Methods).

## Discussion and conclusions

The basic assumption in this work has been that functional sites in proteins are very likely to contain residues that contribute unfavorably to the stability of the native conformation, due to evolutionary selection pressure for optimizing functional efficiency or specificity. This idea has been formulated previously by several authors [25,32,34,38,41], and illustrated in several proteins systems [25-30]. More recently, links have been established between functional sites and unfavorable solvation effects [66] or electrostatic interactions [39]. So far however, the relation between protein residues providing destabilizing contributions and functional sites has not been systematically investigated with rigorous statistical backing.

The present study attempted to fill this gap. It described a procedure for identifying regions in protein structures, containing residues that contribute unfavorably to the thermodynamic stability of the folded state. This stability was assessed from the experimentally determined atomic coordinates on the basis of a classical empirical energy function and standard parameters available in the CHARMM package, augmented with a surface area dependent solvation term. Contributions of individual residues were computed using a thermodynamic cycle that incorporates a simplified model for the unfolded state. Clusters of the most destabilizing residues were identified and extended to include their immediate spatial neighbours, yielding the so-called destabilizing regions defined in this study.

Applying our procedure to a set of 63 high resolution protein crystal structures with well annotated ligand binding sites, but representing the *apo*-form of the protein, we were able to measure the overlap between these annotated sites and the identified destabilizing regions, assess its statistical significance and evaluate the effectiveness of using destabilizing regions for the prediction of ligand binding sites in proteins.

Although our results show that only about 25% of the residues in destabilizing regions, as defined here, belong to ligand binding sites and *vice versa*, this overlap is well above what would be expected by chance (~8%). We find furthermore, that when destabilizing regions are detected, they display statistically significant overlap with at least one known binding site in 77% of proteins examined here.

Another important finding of our analysis is that the extent of overlap between destabilizing regions and binding sites is highly dependent on the type of ligand bound to these sites. More extensive overlap is observed with binding sites for small ligands and polysaccharides whereas the overlap with nucleic acid binding sites is extremely poor. These differences are rationalized by the observation that the binding sites for small ligands and polysaccharides occur mostly in clefts lined with polar residues. Those become partially desolvated, as a result, leading to unfavorable contributions. In contrast, the nucleic acid-binding sites often consist of convex surfaces that are particularly rich in positively charged and polar residues. The latter are hence optimally solvated in absence of the bound nucleotides and therefore provide a favorable energetic contribution. But the relationship between destabilizing regions and functional sites cannot be reduced to geometric features or amino acid composition. For instance, polysaccharide-binding sites are rich in residue types that are rare in destabilizing regions but overlap well with the latter. Likewise, some clefts are not detected as destabilizing (*e.g*. in sphericase) whereas flat and convex regions are occasionally detected as such.

There is little doubt that the occurrence of ligand binding sites in clefts often makes physical and chemical sense. Using the definition of clefts in a protein structure to predict functional sites is however far more challenging. SURFNET is a program that identifies clefts in protein structures. It was claimed by the authors that the largest cleft identified by SURFNET contains the protein binding site in a large majority of cases [18]. Applying SURFNET to our 63 structures and checking the overlap of the largest cleft identified by this procedure with the functional sites defined in our dataset, shows that SURFNET is 'better' at predicting functional site (average sensitivity of 46% compared to 26% with our approach). However SURFNET clefts are usually much larger than the functional sites (see [60]), and therefore tend to include these entirely in addition to including a large number of 'false positive' residues. The PPV of the SURFNET method is consequently much lower (15%) compared to our method (27%). For the same reason, the overlap noted here with destabilizing regions may often not be statistically significant (large size residue patches have a higher probability to overlap with another patch by chance). It was recently shown [58] that the poor specificity of SURFNET could be improved by using it in combination with the conservation-based method CONSURF [6]. Similarly, complementing SURFNET with functional site prediction approaches not based on conservation, like the one presented here, may prove useful for cases where not enough homologues are available.

The energetic criteria used here to define destabilizing regions would also need improvements. These criteria currently rely on standard force fields and approaches, that suffer from many well documented limitations. The representation of electrostatic and solvation effects is far from optimal, although some of us have recently demonstrated that the addition of the simple surface area terms to the CHARMM potential, as done here, is superior to many of the more sophisticated continuum electrostatic models [67]. More importantly, our analysis completely neglects polypeptide chain entropy contributions to the free energy in both the folded and unfolded states, and our model for the unfolded state is extremely crude. Due in part to these limitations we chose not to consider residues with very high temperature factors in our calculations, as the atomic coordinates of those residues are likely to be inaccurate. However, it is well known that flexible regions often tend to be involved in recognition. It is therefore not surprising that our choice not to consider residues with high temperature factors resulted in the elimination of several destabilizing regions that show significant overlap with functional sites (*e.g*. the TcR-binding site of staphylococcal enterotoxin C2 ([PDB:1uns]).

A further factor that most certainly influenced the results of our analysis is the incomplete knowledge that we currently have of the functional sites of proteins on the one hand, and the lack of consistent annotations for the known sites on the other. Even one of the best characterized proteins, such as hen egg white lysozyme, features a myriad of binding and functional sites that have not been annotated and archived in databases. The so-called 'moonlighting' proteins where new binding sites and activities are discovered long after their first function was characterised, illustrate well this point [68]. To compensate at least in part for this shortcoming, the biologically relevant binding sites in the 63 proteins used in our analysis were manually annotated using all available literature evidence. The annotated binding sites are freely available at [53]. Clearly though, much more work is needed in order to produce both more consistent definitions of functional sites and to proceed with their annotations.

Despite the current limitations in using energetic criteria to identify functional sites in proteins, we believe that methods such as those presented here and future improved versions, will play an increasingly important role. Indeed the fact that they do not rely on information on related proteins, as most other methods presently do [6,23], should make them particularly useful for assigning function to proteins with no known relatives, of which a growing number is being currently discovered (meta-genomics projects [69]).

Since functional site prediction methods that use information on sequences and structures of related proteins may also suffer from limitations due to the lack of sequence data, an approach in which such methods are combined with energetic criteria should help improve performance, as previously suggested [23]. Of particular interest are methods that detect spatial clusters of conserved residues, which were shown to greatly improve the performance of functional site prediction [6-9]. Further improvements may be obtained if those methods are combined with the search of spatial clusters of destabilizing residues, as done in this study, instead of considering only individual destabilizing residues [24].

## Methods

### Contributions of individual residues to protein stability

**Figure 4:**
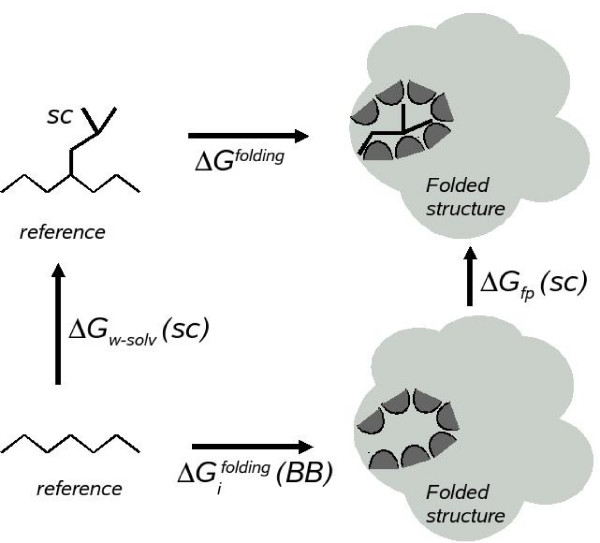
**Thermodynamic cycle for calculating the contribution of a side-chain to the protein folding free energy**. Δ*G*^*folding *^is the folding free energy of the protein in the presence of all amino acids including the one at position *i*. ΔGifolding
 MathType@MTEF@5@5@+=feaafiart1ev1aaatCvAUfKttLearuWrP9MDH5MBPbIqV92AaeXatLxBI9gBaebbnrfifHhDYfgasaacH8akY=wiFfYdH8Gipec8Eeeu0xXdbba9frFj0=OqFfea0dXdd9vqai=hGuQ8kuc9pgc9s8qqaq=dirpe0xb9q8qiLsFr0=vr0=vr0dc8meaabaqaciaacaGaaeqabaqabeGadaaakeaacqqHuoarcqWGhbWrdaqhaaWcbaGaemyAaKgabaGaemOzayMaem4Ba8MaemiBaWMaemizaqMaemyAaKMaemOBa4Maem4zaCgaaaaa@3A36@ (*BB*) is the folding free energy of the protein in the absence of the side chain at position *i*. Δ*G*_*w*-*solv*_(*SC*) is the free energy cost of introducing the side chain of residue *i *into the water solvent. Δ*G*_*fp*_(*SC*) is the free energy cost of introducing the same side chain into the folded protein structure. Δ*G*_*fp*_(*SC*) includes the energy of interaction of the side chain with the surrounding residues in the protein structure, as well as the cost of burying the atoms of both the side chain and the surrounding protein structure.

The contribution of residue i to the folding free energy of the protein is computed as the difference (ΔΔ*G*_*i*_) between the folding free energy in presence and absence of the considered amino acid side chain in position i, using the thermodynamic cycle shown in Figure 4, as follows:

ΔΔGi=ΔGifolding(BB)−ΔGifolding
 MathType@MTEF@5@5@+=feaafiart1ev1aaatCvAUfKttLearuWrP9MDH5MBPbIqV92AaeXatLxBI9gBaebbnrfifHhDYfgasaacH8akY=wiFfYdH8Gipec8Eeeu0xXdbba9frFj0=OqFfea0dXdd9vqai=hGuQ8kuc9pgc9s8qqaq=dirpe0xb9q8qiLsFr0=vr0=vr0dc8meaabaqaciaacaGaaeqabaqabeGadaaakeaacqqHuoarcqqHuoarcqWGhbWrdaWgaaWcbaGaemyAaKgabeaakiabg2da9iabfs5aejabdEeahnaaDaaaleaacqWGPbqAaeaacqWGMbGzcqWGVbWBcqWGSbaBcqWGKbazcqWGPbqAcqWGUbGBcqWGNbWzaaGccqGGOaakcqWGcbGqcqWGcbGqcqGGPaqkcqGHsislcqqHuoarcqWGhbWrdaqhaaWcbaGaemyAaKgabaGaemOzayMaem4Ba8MaemiBaWMaemizaqMaemyAaKMaemOBa4Maem4zaCgaaaaa@52FD@

with ΔGifolding
 MathType@MTEF@5@5@+=feaafiart1ev1aaatCvAUfKttLearuWrP9MDH5MBPbIqV92AaeXatLxBI9gBaebbnrfifHhDYfgasaacH8akY=wiFfYdH8Gipec8Eeeu0xXdbba9frFj0=OqFfea0dXdd9vqai=hGuQ8kuc9pgc9s8qqaq=dirpe0xb9q8qiLsFr0=vr0=vr0dc8meaabaqaciaacaGaaeqabaqabeGadaaakeaacqqHuoarcqWGhbWrdaqhaaWcbaGaemyAaKgabaGaemOzayMaem4Ba8MaemiBaWMaemizaqMaemyAaKMaemOBa4Maem4zaCgaaaaa@3A36@ representing the folding free energy of the protein in the presence of all the amino acids including that at position i, and ΔGifolding
 MathType@MTEF@5@5@+=feaafiart1ev1aaatCvAUfKttLearuWrP9MDH5MBPbIqV92AaeXatLxBI9gBaebbnrfifHhDYfgasaacH8akY=wiFfYdH8Gipec8Eeeu0xXdbba9frFj0=OqFfea0dXdd9vqai=hGuQ8kuc9pgc9s8qqaq=dirpe0xb9q8qiLsFr0=vr0=vr0dc8meaabaqaciaacaGaaeqabaqabeGadaaakeaacqqHuoarcqWGhbWrdaqhaaWcbaGaemyAaKgabaGaemOzayMaem4Ba8MaemiBaWMaemizaqMaemyAaKMaemOBa4Maem4zaCgaaaaa@3A36@ (*BB*) representing the folding free energy of the entire protein in absence of the sidechain at position i. ΔΔ*G*_*i *_hence takes into account the total free energy cost of desolvating in part or in whole the amino acid itself, as well as the cost of the partial desolvation of neighbouring residues and the vacuum interaction terms of the considered residue with all surrounding atoms. ΔGifolding
 MathType@MTEF@5@5@+=feaafiart1ev1aaatCvAUfKttLearuWrP9MDH5MBPbIqV92AaeXatLxBI9gBaebbnrfifHhDYfgasaacH8akY=wiFfYdH8Gipec8Eeeu0xXdbba9frFj0=OqFfea0dXdd9vqai=hGuQ8kuc9pgc9s8qqaq=dirpe0xb9q8qiLsFr0=vr0=vr0dc8meaabaqaciaacaGaaeqabaqabeGadaaakeaacqqHuoarcqWGhbWrdaqhaaWcbaGaemyAaKgabaGaemOzayMaem4Ba8MaemiBaWMaemizaqMaemyAaKMaemOBa4Maem4zaCgaaaaa@3A36@ is computed as previously described [51]:

Δ*G *^*folding *^= *G *^*folded *^- *G *^*reference*^

Where *G *^*folded *^is the protein free energy in the folded state and *G *^*reference *^the free energy in a reference state, which is used as a model for the protein unfolded state. The free energy of the folded state is then expressed as an effective energy, which is the sum of the following terms [51]:

*G *^*folded *^= *E *^*conformation *^+ *G *^*solvation*^

*E*^*conformation *^is the classical conformational energy computed using the CHARMM 22 force field [70] which is expressed as a sum of pairwise contributions, and uses a full atom representation. *G *^*solvation*^represents the solvation free energy, computed using an empirical atomic solvation model [71] (see references [36,51] for further details). In these calculations the electrostatic term is computed using a dielectric constant of 8 and a switching function operating between 6–7 Å

The free energy of the reference state *G *^*reference *^is calculated as the sum of the free energy contributions of isolated amino acids:

Greference=∑iGireference
 MathType@MTEF@5@5@+=feaafiart1ev1aaatCvAUfKttLearuWrP9MDH5MBPbIqV92AaeXatLxBI9gBaebbnrfifHhDYfgasaacH8akY=wiFfYdH8Gipec8Eeeu0xXdbba9frFj0=OqFfea0dXdd9vqai=hGuQ8kuc9pgc9s8qqaq=dirpe0xb9q8qiLsFr0=vr0=vr0dc8meaabaqaciaacaGaaeqabaqabeGadaaakeaacqWGhbWrdaahaaWcbeqaaiabdkhaYjabdwgaLjabdAgaMjabdwgaLjabdkhaYjabdwgaLjabd6gaUjabdogaJjabdwgaLbaakiabg2da9maaqafabaGaem4raC0aa0baaSqaaiabdMgaPbqaaiabdkhaYjabdwgaLjabdAgaMjabdwgaLjabdkhaYjabdwgaLjabd6gaUjabdogaJjabdwgaLbaaaeaacqWGPbqAaeqaniabggHiLdaaaa@4D70@

Where i are the isolated amino acids, modelled by a standard dipeptide unit with N-acetyl-N'-methylamide backbone, and the sum is performed over the sequence of the protein. As for the folded state, *G*^*reference *^is expressed as a sum of two terms:

Gireference=Eiconformation+Gisolvation
 MathType@MTEF@5@5@+=feaafiart1ev1aaatCvAUfKttLearuWrP9MDH5MBPbIqV92AaeXatLxBI9gBaebbnrfifHhDYfgasaacH8akY=wiFfYdH8Gipec8Eeeu0xXdbba9frFj0=OqFfea0dXdd9vqai=hGuQ8kuc9pgc9s8qqaq=dirpe0xb9q8qiLsFr0=vr0=vr0dc8meaabaqaciaacaGaaeqabaqabeGadaaakeaacqWGhbWrdaqhaaWcbaGaemyAaKgabaGaemOCaiNaemyzauMaemOzayMaemyzauMaemOCaiNaemyzauMaemOBa4Maem4yamMaemyzaugaaOGaeyypa0Jaemyrau0aa0baaSqaaiabdMgaPbqaaiabdogaJjabd+gaVjabd6gaUjabdAgaMjabd+gaVjabdkhaYjabd2gaTjabdggaHjabdsha0jabdMgaPjabd+gaVjabd6gaUbaakiabgUcaRiabdEeahnaaDaaaleaacqWGPbqAaeaacqWGZbWCcqWGVbWBcqWGSbaBcqWG2bGDcqWGHbqycqWG0baDcqWGPbqAcqWGVbWBcqWGUbGBaaaaaa@5FC9@

where Eiconformation
 MathType@MTEF@5@5@+=feaafiart1ev1aaatCvAUfKttLearuWrP9MDH5MBPbIqV92AaeXatLxBI9gBaebbnrfifHhDYfgasaacH8akY=wiFfYdH8Gipec8Eeeu0xXdbba9frFj0=OqFfea0dXdd9vqai=hGuQ8kuc9pgc9s8qqaq=dirpe0xb9q8qiLsFr0=vr0=vr0dc8meaabaqaciaacaGaaeqabaqabeGadaaakeaacqWGfbqrdaqhaaWcbaGaemyAaKgabaGaem4yamMaem4Ba8MaemOBa4MaemOzayMaem4Ba8MaemOCaiNaemyBa0MaemyyaeMaemiDaqNaemyAaKMaem4Ba8MaemOBa4gaaaaa@3FD1@ and Gisolvation
 MathType@MTEF@5@5@+=feaafiart1ev1aaatCvAUfKttLearuWrP9MDH5MBPbIqV92AaeXatLxBI9gBaebbnrfifHhDYfgasaacH8akY=wiFfYdH8Gipec8Eeeu0xXdbba9frFj0=OqFfea0dXdd9vqai=hGuQ8kuc9pgc9s8qqaq=dirpe0xb9q8qiLsFr0=vr0=vr0dc8meaabaqaciaacaGaaeqabaqabeGadaaakeaacqWGhbWrdaqhaaWcbaGaemyAaKgabaGaem4CamNaem4Ba8MaemiBaWMaemODayNaemyyaeMaemiDaqNaemyAaKMaem4Ba8MaemOBa4gaaaaa@3BDA@ are the contributions from conformational and solvation energies, respectively. Calculation of the two energy terms in Eq. 6 involves computing the Boltzmann averages of the conformational and solvation energies over all possible side chain conformations of amino acid i. The same force-field is used as for the folded state calculations.

Prior to computing the energies in Eqs 4 and 6, hydrogen positions are added using the HBUILD command in charmm [72]. Histidine protonation is assigned on the basis of distance to neighbouring residues. The resulting structures are relaxed by applying 50 steps of steepest descent energy minimisation. The biologically meaningful quaternary structures are obtained with PQS [73], and manually verified with information from the PDB file and from the literature whenever available.

### Defining clusters of destabilizing residues

The contribution of individual residues to the protein folding free energy is computed as detailed above and the values are ranked in ascending order starting with the most unfavorable contribution. Destabilizing residues are defined as the 28% residues with largest unfavorable contributions to stability in each protein, over the total ranked list of residues. A subset of "highly destabilizing" residues is defined as the 5% of the residues with the most destabilizing contributions. The precise values for these two thresholds were derived, as described in section on parameter fitting (below).

**Figure 5:**
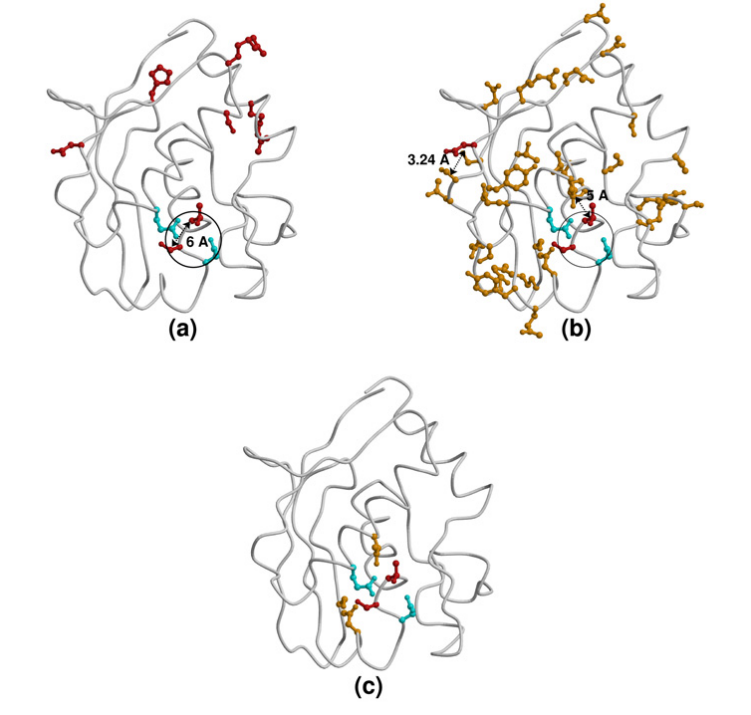
**Destabilizing regions detection procedure**. (*a*) clustering of highly destabilizing residues (red) that are less than 9.0 Å apart. (*b*) addition of destabilizing residues (orange) that are within 6.0 Å of a destabilizing residue already present in a destabilizing region. (*c*) Final result. Only the destabilizing regions larger than 4 residues are considered. In (*a*) and (*b*) are represented residues (cyan), destabilizing or not, which are enclosed in a sphere centered on a pair of destabilizing residues and therefore added to the destabilizing region (see text for more details).

Destabilizing regions are detected using a 2-steps distance-based spatial clustering procedure illustrated at Figure 5 with pdb entry 1c7k. Each highly destabilizing residue is the seed of a destabilizing region. The highly destabilizing residues are clustered if they are less than 9.0 Å apart. Then, any destabilizing residue is added to a cluster if it is within 6.0 Å of a destabilizing residue already present in the cluster. In addition, when destabilizing residues are paired, a sphere, whose center is the center-of-mass of the pair and whose diameter is 75% of the distance between the 2 residues, is considered; any residue enclosed in that sphere, whether destabilizing or not, is added to the corresponding destabilizing region. Finally, destabilizing regions with less than 4 residues are discarded. This represents the size of the smallest binding site in the dataset.

The centre-of-mass of the residue side chain, excluding *C*_*β*_, is used in the calculation of all distances, with the exception of Gly and Ala, where the *C*_*α *_and *C*_*β *_coordinates are used, respectively. This approach is inspired by a method used to identify clusters of conserved residues [9].

The software DESITE for identifying destabilizing regions from the atomic coordinates is available upon request from the authors. A license to the CHARMM package is required.

### Parameter adjustments

Our procedure has a total of 4 adjustable parameters. These are the fractions of residues with unfavorable contributions to stability used to define the destabilizing and highly destabilizing residues, and the distance thresholds used to group highly destabilizing residues and destabilizing residues into the same destabilizing region.

The values of these parameters were obtained as follows. We first defined "reasonable" ranges of values according to the physical meaning of the parameters (*e.g*. distance parameters cannot be too large or the destabilizing regions would consist of the entire protein). We used the following "acceptable" ranges: the proportion of destabilizing residues is varied between 6 and 30%, whereas that of highly destabilizing residues is varied between 1 and 10%, and the distance for grouping 2 highly destabilizing residues is varied between 8 and 12 Å whereas that for adding a destabilizing residue to a cluster is varied between 5 and 10 Å. Values were changed in intervals of 1 (Å or %) within these ranges. Destabilizing regions obtained with all possible values combinations (with logical restrictions, *i. e*. the proportion of highly destabilizing residues must be smaller than that of destabilizing residues) were compared with known binding sites in 7 proteins selected as described in the section on Protein datasets. The pdb identifiers of the *apo*-structures of these proteins used for parametrisation are 1bn6, 1c5h, 1e5m, 1glo, 1hl4, 1ogh and 1ojx. We selected the combination of values that yielded the best overall prediction accuracy with regard to the known functional sites in these proteins.

### Filtering criteria

Not all identified clusters of destabilizing residues were considered for further analysis. Positions of atoms having high temperature factors (B factors) are considered as inaccurate, and could yield destabilizing contributions due their inaccuracies. Regions where more than half of the destabilizing residues either have a high average B factor or are located within 5 Å of such residues are therefore not analyzed. The average B factor of a residue is considered as "high" if it is larger than the average B factors taken over all residues in the protein plus two standard deviations. Regions where the majority of the residues have alternate conformations are also discarded because they may yield destabilizing contributions to be due to incompatible combinations of the alternate conformations. In homo multimeric proteins a destabilizing region is discarded if it is not detected in all subunits.

### Protein datasets

From the February 2005 release of the PDB [44], we selected the subset of x-ray structures released after November 3rd 1999, with a resolution better than 2.4 Å, a R-value better than 0.20, no residues with missing coordinates except at the termini, and a SITE record in the pdb file. To eliminate structures with bound ligands (potential *holo*-forms), we filtered out entries with nucleic-acid chains or small ligands (HET-groups), and those that were neither protein monomers or homomultimers. Applying these drastic filters, and removing redundancy, using PISCES [74] with a 25% sequence identity cutoff, resulted in a set of only 7 structures. Those were used as our learning set to derive the values of the 4 adjustable parameters as described above.

To build our test set of *apo*-structures, we relaxed the above-mentioned filter on HET-groups to accept structures with HET-groups of 5 atoms or less, but verifying in all cases that these were not the biologically relevant ligands. To guarantee we could compare the predictions calculated on these *apo*-structures with the true functional sites for all proteins in the dataset, we excluded the proteins for which we did not find information on functionally important residues from any of the sources described in the section "definition of known binding sites" (see below).

Due to the SITE record filter, this dataset contained mostly enzymes. In order to include non-enzymes as well, we expanded the set by releasing the SITE record filter, while allowing only non-enzymatic proteins to be added. These structures were also filtered to remove entries with ligands, but included those for which a *holo*-structure, another structure of the same protein with its biologically relevant ligand, was also available (this was done using the RELATED record of the pdb file). The *holo*-structures were used to aid the functional site definition.

Redundancy was removed with a 25% sequence identity cutoff [74]. The final test set contains 63 proteins, comprising 35 enzymatic and 28 non-enzymatic proteins. In contrast, the learning set contains 7 proteins, all of which are enzymes.

### Definition of known binding sites

Known binding sites are defined on the basis of structural and biochemical information. When one or several *holo*-structures of the protein are available, we define a residue as ligand binding when at least two of its atoms are within a 6 Å distance from a biologically relevant ligand in the (ensemble of) *holo*-structures, ignoring hydrogens. To this the contents of the SITE record is added, manually checking that it contains biologically relevant information. The binding site definition is complemented by site-directed mutagenesis and chemical modification data, whenever relevant for the function of the protein, obtained by manually searching the available literature. If the structure of a protein in complex with a biologically relevant ligand is not available, the functional site is defined from literature only. We consider information from close homologs when there is evidence that the function is conserved. Out of the 63 proteins in the validation dataset, 49 have a binding site based on structural information only, *i.e*., where literature search did not add any residue, 10 have a binding site derived from a combination of literature searches and presence of close homolog complexes in the pdb, and for 7 proteins the binding site definition is based on literature search only. For these 7 proteins the known binding site does not form a continuous surface patch.

Protein-bound metal ions can have no other function than to stabilise the protein structure, or they can be directly implicated in the molecular function, as is the case for 8 proteins in the dataset. Metal-binding sites are considered only if the metal is known to be important for function, and if in addition it is absent from the *apo*-structure used in the energy calculations.

The descriptions of the known binding sites, with the literature citations are available at [53].

### Evaluating the overlap between destabilizing regions and functional sites

To evaluate the overlap between destabilizing regions and known binding sites, the residues in each site (and region) are compared and the number of residues in common is computed. The number of overlapping residues expected by chance for each binding site – destabilizing region pair is computed using the hypergeometric distribution and from it a statistical significance value (P-value) is computed for the observed overlap, as implemented in the software Compare-Classes of the RSA-tools package [75]. When multiple destabilizing regions are detected in a given protein, they are grouped together as a single one for computing the P-value. For a destabilizing region and a functional site containing *a *and *b *residues, respectively, the probability of finding exactly *c *common residues between them is

P(X=c)=(Ccb∗Ca−cn−b)Can
 MathType@MTEF@5@5@+=feaafiart1ev1aaatCvAUfKttLearuWrP9MDH5MBPbIqV92AaeXatLxBI9gBaebbnrfifHhDYfgasaacH8akY=wiFfYdH8Gipec8Eeeu0xXdbba9frFj0=OqFfea0dXdd9vqai=hGuQ8kuc9pgc9s8qqaq=dirpe0xb9q8qiLsFr0=vr0=vr0dc8meaabaqaciaacaGaaeqabaqabeGadaaakeaacqWGqbaucqGGOaakcqWGybawcqGH9aqpcqWGJbWycqGGPaqkcqGH9aqpdaWcaaqaaiabcIcaOiabdoeadnaaDaaaleaacqWGJbWyaeaacqWGIbGyaaGccqGHxiIkcqWGdbWqdaqhaaWcbaGaemyyaeMaeyOeI0Iaem4yamgabaGaemOBa4MaeyOeI0IaemOyaigaaOGaeiykaKcabaGaem4qam0aa0baaSqaaiabdggaHbqaaiabd6gaUbaaaaaaaa@4706@

where Cxy
 MathType@MTEF@5@5@+=feaafiart1ev1aaatCvAUfKttLearuWrP9MDH5MBPbIqV92AaeXatLxBI9gBaebbnrfifHhDYfgasaacH8akY=wiFfYdH8Gipec8Eeeu0xXdbba9frFj0=OqFfea0dXdd9vqai=hGuQ8kuc9pgc9s8qqaq=dirpe0xb9q8qiLsFr0=vr0=vr0dc8meaabaqaciaacaGaaeqabaqabeGadaaakeaacqWGdbWqdaqhaaWcbaGaemiEaGhabaGaemyEaKhaaaaa@30DC@ is the binomial coefficient. The probability of observing at least *c *residues in common by chance is given by

P(X≥c)=1−∑i=0c−1P(X=i)
 MathType@MTEF@5@5@+=feaafiart1ev1aaatCvAUfKttLearuWrP9MDH5MBPbIqV92AaeXatLxBI9gBaebbnrfifHhDYfgasaacH8akY=wiFfYdH8Gipec8Eeeu0xXdbba9frFj0=OqFfea0dXdd9vqai=hGuQ8kuc9pgc9s8qqaq=dirpe0xb9q8qiLsFr0=vr0=vr0dc8meaabaqaciaacaGaaeqabaqabeGadaaakeaacqWGqbaucqGGOaakcqWGybawcqGHLjYScqWGJbWycqGGPaqkcqGH9aqpcqaIXaqmcqGHsisldaaeWbqaaiabdcfaqjabcIcaOiabdIfayjabg2da9iabdMgaPjabcMcaPaWcbaGaemyAaKMaeyypa0JaeGimaadabaGaem4yamMaeyOeI0IaeGymaedaniabggHiLdaaaa@45EA@

The quality of the overlap is also measured with the Sensitivity and Positive Predictive Value (PPV) :

Sensitivity=TP(TP+FN)
 MathType@MTEF@5@5@+=feaafiart1ev1aaatCvAUfKttLearuWrP9MDH5MBPbIqV92AaeXatLxBI9gBaebbnrfifHhDYfgasaacH8akY=wiFfYdH8Gipec8Eeeu0xXdbba9frFj0=OqFfea0dXdd9vqai=hGuQ8kuc9pgc9s8qqaq=dirpe0xb9q8qiLsFr0=vr0=vr0dc8meaabaqaciaacaGaaeqabaqabeGadaaakeaacqWGtbWucqWGLbqzcqWGUbGBcqWGZbWCcqWGPbqAcqWG0baDcqWGPbqAcqWG2bGDcqWGPbqAcqWG0baDcqWG5bqEcqGH9aqpdaWcaaqaaiabdsfaujabdcfaqbqaaiabcIcaOiabdsfaujabdcfaqjabgUcaRiabdAeagjabd6eaojabcMcaPaaaaaa@467D@

PPV=TP(TP+FP)
 MathType@MTEF@5@5@+=feaafiart1ev1aaatCvAUfKttLearuWrP9MDH5MBPbIqV92AaeXatLxBI9gBaebbnrfifHhDYfgasaacH8akY=wiFfYdH8Gipec8Eeeu0xXdbba9frFj0=OqFfea0dXdd9vqai=hGuQ8kuc9pgc9s8qqaq=dirpe0xb9q8qiLsFr0=vr0=vr0dc8meaabaqaciaacaGaaeqabaqabeGadaaakeaacqWGqbaucqWGqbaucqWGwbGvcqGH9aqpdaWcaaqaaiabdsfaujabdcfaqbqaaiabcIcaOiabdsfaujabdcfaqjabgUcaRiabdAeagjabdcfaqjabcMcaPaaaaaa@3ACF@

Where *TP *(true positives) is the number of residues correctly predicted as part of the binding site; *FP *(false positives) is the number of residues incorrectly predicted as part of the binding site, and *FN *(false negatives) is the number of residues incorrectly predicted as not part of the binding site.

*Sensitivity *is the proportion of residues in a known binding site that are found in the predicted sites (*i.e*. the destabilizing regions), and the *PPV *is the proportion of predicted sites residues that are part of a known binding site.

## Authors' contributions

All authors participated in the design of the study. BHD performed the analysis and drafted the manuscript. MFL and SJW provided feedback throughout the project, and SJW directed the work. All authors read and approved the final manuscript.
